# Salivary Gland Hypofunction in *tyrosylprotein sulfotransferase-2* Knockout Mice Is Due to Primary Hypothyroidism

**DOI:** 10.1371/journal.pone.0071822

**Published:** 2013-08-07

**Authors:** Andrew D. Westmuckett, Joseph C. Siefert, Yasvir A. Tesiram, David M. Pinson, Kevin L. Moore

**Affiliations:** 1 Cardiovascular Biology Research Program, Oklahoma Medical Research Foundation, Oklahoma City, Oklahoma, United States of America; 2 Department of Cell Biology, University of Oklahoma Health Sciences Center, Oklahoma City, Oklahoma, United States of America; 3 Centre for Advanced Imaging, the University of Queensland, Brisbane, Australia; 4 Department of Pathology, University of Illinois College of Medicine, Peoria, Illinois, United States of America; 5 Department of Medicine, University of Oklahoma Health Sciences Center, Oklahoma City, Oklahoma, United States of America; 6 Oklahoma Center of Medical Glycobiology, University of Oklahoma Health Sciences Center, Oklahoma City, Oklahoma, United States of America; University of Michigan Medical School, United States of America

## Abstract

**Background:**

Protein-tyrosine sulfation is a post-translational modification of an unknown number of secreted and membrane proteins mediated by two known Golgi tyrosylprotein sulfotransferases (TPST-1 and TPST-2). We reported that *Tpst2-/-* mice have mild-moderate primary hypothyroidism, whereas *Tpst1-/-* mice are euthyroid. While using magnetic resonance imaging (MRI) to look at the thyroid gland we noticed that the salivary glands in *Tpst2-/-* mice appeared smaller than in wild type mice. This prompted a detailed analysis to compare salivary gland structure and function in wild type, *Tpst1-/-*, and *Tpst2* -/- mice.

**Methodology/Principal Findings:**

Quantitative MRI imaging documented that salivary glands in *Tpst2-/-* females were ^≈^ 30% smaller than wild type or *Tpst1-/-* mice and that the granular convoluted tubules in *Tpst2-/-* submandibular glands were less prominent and were almost completely devoid of exocrine secretory granules compared to glands from wild type or *Tpst1-/-* mice. In addition, pilocarpine–induced salivary flow and salivary α-amylase activity in *Tpst2-/-* mice of both sexes was substantially lower than in wild type and *Tpst1-/-* mice. Anti-sulfotyrosine Western blots of salivary gland extracts and saliva showed no differences between wild type, *Tpst1-/-*, and *Tpst2-/-* mice, suggesting that the salivary gland hypofunction is due to factor(s) extrinsic to the salivary glands. Finally, we found that all indicators of hypothyroidism (serum T4, body weight) and salivary gland hypofunction (salivary flow, salivary α-amylase activity, histological changes) were restored to normal or near normal by thyroid hormone supplementation.

**Conclusions/Significance:**

Our findings conclusively demonstrate that low body weight and salivary gland hypofunction in *Tpst2-/-* mice is due solely to primary hypothyroidism.

## Introduction

Tyrosine *O*-sulfation is a posttranslational modification catalysed by tyrosylprotein sulfotransferases (TPSTs) that mediate the transfer of a sulfuryl group from the sulfate donor 3’-phosphoadenosine 5’-phosphosulfate (PAPS) to the hydroxyl group of peptidyl-tyrosine to form a tyrosine *O* ^4^-sulfate ester and 3’, 5’-ADP [1]. Although tyrosine sulfation was first described almost 60 years ago [2], the first TPSTs were not purified and cloned until 1998 [3–5]. Most animal genomes appear to have two TPST genes, although only one *Tpst* gene is apparent in *Drosophila melanogaster* [1,6]. More recently, the first plant TPST was purified and cloned in *Arabidopsis thaliana* by Komori et al. in 2009 [7]. Subsequently, a TPST was identified in the Gram-negative bacterium *Xanthomonas* *oryzae* pv*. Oryzae* [8].

The two mammalian enzymes are known as TPST-1 and TPST-2. The subcellular localization in the trans-Golgi network and the widespread tissue and cellular distribution of the two enzymes has been well documented [1]. Several dozen tyrosine-sulfated proteins, mostly of animal origin, have been described, many of which play important roles in inflammation, hemostasis, immunity, and other processes, including certain adhesion molecules, G-protein coupled receptors, coagulation factors, serpins, extracellular matrix proteins, hormones, and others [1,9,10]. Although the importance of protein-tyrosine sulfation in protein–protein interactions has become generally accepted, there is still much that is unknown about the role of tyrosine sulfation in protein function and physiology.

To gain insights into the role of tyrosine sulfation *in vivo*, we generated *Tpst1-/-* and *Tpst2-/-* knockout mice and have characterized them over the past decade. *Tpst1-/-* mice have a very mild phenotype [11]. They appear healthy but have ^≈^ 5% lower average body weight than Tpst1+/+ controls. In addition, we showed that although fertility of *Tpst1-/-* males and females *per se* is normal, *Tpst1-/-* females have significantly smaller litters due to fetal death between 8.5 and 15.5 days post coitum. The mechanism for the fetal loss remains unexplored.

In contrast, we have reported two very interesting phenotypes in *Tpst2-/-* mice. First, *Tpst2-/-* males are sterile [12]. *Tpst2-/-* males are eugonadal and *Tpst2* null sperm are normal in number, morphology, and motility in normal media and appear to capacitate and undergo acrosomal exocytosis normally. However, *Tpst2* null sperm are defective in motility in viscous medium and in their ability to fertilize cumulus-enclosed eggs. Additionally, *in vitro* fertilization experiments with zona pellucida-free eggs revealed that *Tpst2-/-* null sperm adhered poorly to the egg plasma membrane, but somewhat paradoxically appeared to have an increased extent of sperm-egg fusion. We later reported the increased sperm-egg fusion was not due to a failure of *Tpst2* null sperm to trigger establishment of the egg membrane block to polyspermy [13]. We also found that testicular sperm from *Tpst2-/-* mice sperm express ADAM6 and ADAM3, but unlike wild type sperm, ADAM6 and ADAM3 expression is lost on epididymal sperm from the knockout. Loss of ADAM3 is strongly associated with male infertility in mice with targeted deletion of *Adam1a*, *Adam2*, *Clgn* (calmegin), and *Calr3* (calsperin) [14–17].

We also reported that *Tpst2-/-* mice have mild-moderate primary hypothyroidism, whereas *Tpst1-/-* mice are euthyroid [18] consistent with the observations by Sasaki et al. that a spontaneous mutation in the *Tpst2* gene is responsible for an autosomal recessive form of primary hypothyroidism in the *grt*/*grt* mouse [19].

While imaging the thyroid gland in *Tpst2-/-* mice using magnetic resonance imaging (MRI) we stumbled onto a third interesting phenotype. We noticed that the salivary glands in *Tpst2-/-* mice appeared smaller than in wild type mice. This finding prompted the current study, in which we describe the results of a detailed comparative analysis of the salivary gland structure and function in wild type, *Tpst1-/-*, and *Tpst2* -/- mice. Our studies demonstrate that *Tpst2-/-*, but not *Tpst1-/-* mice, have salivary gland hypofunction and that salivary gland hypofunction is due solely to primary hypothyroidism.

## Methods

### Ethics statement

All procedures involving vertebrate animals were reviewed and approved by the Institutional Animal Care and Use Committee at the Oklahoma Medical Research Foundation (Protocols 10-19) and were performed in accordance with the 8th Edition of the *Guide for the Care and Use of Laboratory Animals* (NRC 2011).

### Animals

*Tpst1-/-* (Tpst1^tm1Klm^, MGI:2183366) and *Tpst2-/-* (Tpst2^tm1Klm^, MGI:3512111) mice on the 129S6/SvEvTac background were generated, characterized, and housed as previously described [11,12]. Genotypes were confirmed by PCR for the presence of wild type and mutant alleles at the *Tpst1* and *Tpst2* loci as previously described [11,12].

### Antibodies

The anti-sulfotyrosine monoclonal antibody (mAb) PSG2 (human IgG4-λ) was characterized and purified as previously described [20]. An isotype control human IgG4-λ mAb (#I4764) and horseradish peroxidase (HRP)-conjugated anti-human IgG (#A0170) were purchased from Sigma-Aldrich.

### Magnetic resonance imaging

**Figure 1:**
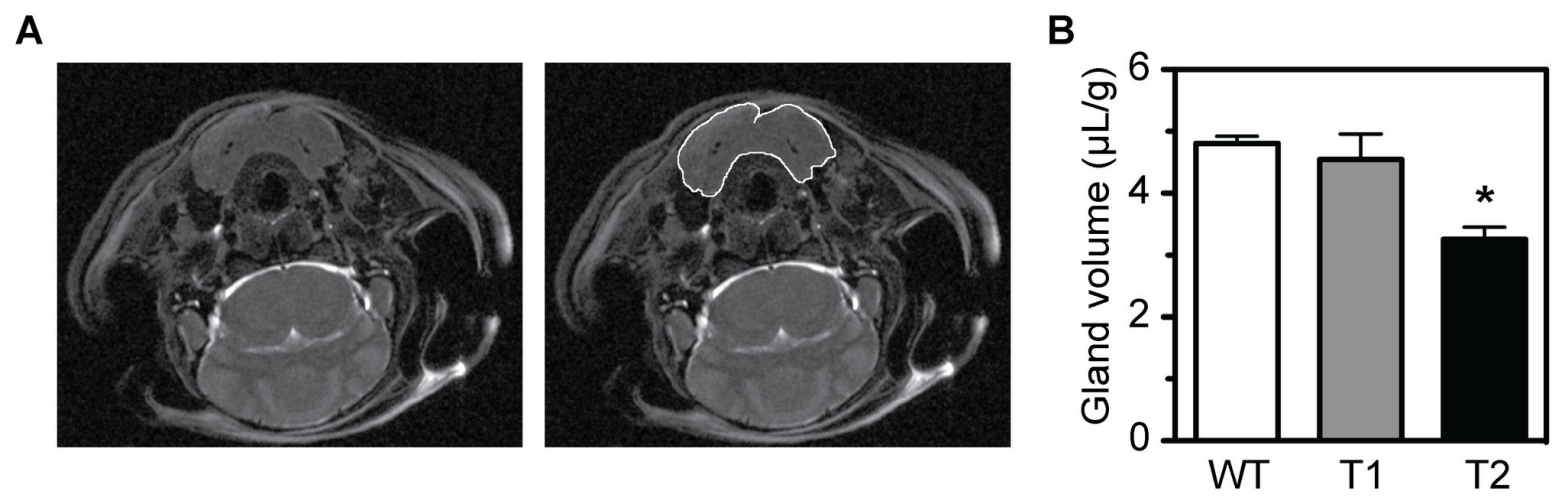
Magnetic resonance imaging. Contiguous coronal images were collected from wild type (WT), *Tpst1-/-* (T1), and *Tpst2-/-* (T2) females (n = 3). The tissue region comprising the right and left sublingual and submandibular glands (A) were traced and an area per slice was calculated and a total volume was determined which was then normalized to body weight in grams as described in Methods (B). Results are expressed as mean ± S.E.M. Statistical differences between groups were tested using unpaired, two-tailed *t*-tests with equal sample variance and an α ≤ 0.05. Salivary gland volume of *Tpst2* -/- mice was significantly smaller than wild type (*p* = 0.002) and *Tpst1-/-* (*p* = 0.04), respectively (indicated by an asterisk). However, the salivary gland volumes of wild type and *Tpst1-/-* mice were not statistically different (*p* = 0.57).

Animals were imaged at the Advanced Magnetic Resonance Center at the Oklahoma Medical Research Foundation using a Bruker AVANCE III, 11.74 Tesla NMR spectrometer (Bruker BioSpin MRI Gmbh, Ettlingen, Germany) equipped with a 30 mm diameter quadrature coil resonator and micro-imaging apparatus. Each animal was anesthetized with 1% isoflurane at 0.45 L/min oxygen and anesthesia was adjusted to maintain a constant respiratory rate that was monitored throughout the experiment, using an abdominal pneumatic pillow (SA Instruments, Inc., Stony Brook, NY). Body temperature was maintained at 37^°^ C (spectrometer controlled). Animals were secured in an imaging cradle and then introduced into the spectrometer. Transverse images were collected using a Turbo-RARE (rapid acquisition with relaxation enhancement) sequence [TR (repetition time) = 3.5 s, TE (echo time) = 14 ms, effective echo time = 48 ms, RARE factor = 8, averages = 8, refocusing flip angle = 180^°^, with fat suppression, magnetization transfer and motion suppression]. Image segments were collected using a 25.6 mm x 25.6 mm field of view, slice thickness of 0.5 mm, and a 384 x 384 matrix, resulting in a 67 x 67 mm^2^ in-plane resolution. Images were processed using the Paravision 5.0 ROI (region-of-interest) tool (Bruker Biospin) where the tissue region comprising the right and left sublingual and submandibular glands were traced (Figure **1**) and an area (cm^2^) per slice (0.5 mm thick) was calculated and a total volume (µL) was determined. The parotid glands were not readily discernible using MRI. One individual that was blinded to the genotype of the animal performed the gland volume determinations.

### Saliva collection

Mice were fasted for 4 h with water provided *ad libitum*. Following anesthesia, mice received 0.8 µg/g body weight of pilocarpine-HCl in 0.9% sodium chloride *i.p.* and held in a support cradle head-down. Saliva was drained from the base of the upper incisors through a PTFE capillary tube (i.d. 0.8 mm) under a slight vacuum over a 15 min period into a 1.5 mL centrifuge tube. Saliva volume was measured and samples were stored at -86^°^ C for further analysis.

### Salivary amylase and protein assays

Salivary α-amylase was determined using a kinetic chromogenic assay that measures the hydrolysis of 2-chloro-*p*-nitrophenol maltotrisose (Salimetrics α-amylase assay kit, State College, PA). Frozen saliva samples were thawed at room temperature and clarified by centrifugation (1,500 *g*, 15 min). Amylase substrate was pre-warmed to 37^°^ C and added to diluted samples. The rate of substrate hydrolysis was determined at 405 nm using a FLUOstar Omega microplate reader (Molecular Devices Corp). Amylase activity in each sample was calculated according to the instruction of the supplier. Rates of substrate hydrolysis were referenced to values obtained from α-amylase standards. Salivary protein concentrations were determined by bicinchoninic acid (BCA) assay (Pierce). Salivary α-amylase activity was normalized to protein content by dividing salivary amylase activity by protein concentration.

### Histology

For standard histology, tissues were fixed in 10% phosphate buffered zinc formalin and processed for wax embedding. Sections (5 µm) were cut and stained with Masson’s trichrome. Slides were viewed using a Nikon Eclipse E800 microscope and images were captured using a Nikon Sight DS-Fi1 digital camera.

For semi-thin resin sections, tissues were cut into 1-mm^3^ pieces and fixed with 2% glutaraldehyde in 0.1 M sodium cacodylate buffer for 1 h at room temperature. Tissues were washed for 15 min in 2 changes of 0.1 M sodium cacodylate buffer and post-fixed in 1% osmium tetroxide in 0.1 M sodium cacodylate buffer for 1 h at room temperature. Tissues were washed 3 times for 15 min in distilled water and then dehydrated through a graded ethanol series. Tissues were then transferred to LR White (Fluka Analytical) for 1 h and then into fresh LR White for 6 h. Tissues were placed at the base of gelatin capsules. Capsules were filled with fresh LR White and capped to exclude air. Capsules were then incubated in a 50°C oven for two days to polymerize the resin. Polymerized tissues were sectioned at 0.5 µm on a Leica EM UC6 Ultracut Microtome and floated onto drops of 1% toluidine blue in 1% sodium borate at 50°C for 5 min. Stained sections were floated on distilled water, transferred to a glass slide, dried at 50°C, and mounted in VectaMount (Vector Laboratories).

### Thyroid supplementation

*Tpst2+/-* males and females were conditioned to control chow (Harlan, 8664 Teklad F6 rodent diet) or control chow supplemented with 0.025% grade III porcine thyroid powder (Sigma, #T6384) *ad libitum* for 3 weeks. Mating pairs of *Tpst2+/-* males and females were then established and fed the control or thyroid supplemented diets through pregnancy and birth until offspring were weaned. At P21, pups were weaned and maintained on the same diet until they were sacrificed at 15 weeks of age.

### Thyroid function test

Blood was collected at 5, 10, and 15 weeks of age. Serum total T4 assays were performed in duplicate using Coat-A-Count RIA kits (Siemens Nederland, Den Haag, Netherlands).

### SDS-PAGE and Western blotting

Tissues were homogenized in ice-cold 100 mM NaCl, 20 mM MOPS, pH 7.5 supplemented with 10 µg/mL leupeptin, 10 µg/mL antipain, and 1 mM benzamidine using a Dounce homogenizer. The homogenates were centrifuged (2000 *g*, 10 min) to obtain a post-nuclear supernatant. The supernatant was collected and stored at -80° C.

Samples were electrophoresed on NuPAGE^®^ 4-12% Bis-Tris SDS-polyacrylamide gels with 2-(N-morpholino) ethanesulfonic (MES) SDS running buffer (Invitrogen). In all cases, 15 pmoles of human heparin cofactor II, a known tyrosine-sulfated protein (Haematologic Technologies), was run as a positive control [21]. Proteins were electroblotted onto BA85 Protran™ 0.45 µm nitrocellulose membranes (Whatman) or Immobilon™ PVDF membranes (Millipore) using a Transblot SD semi-dry transfer cell (Bio-Rad). Membranes were blocked with 5% non-fat dry milk in Tris-buffered saline containing 0.1% Tween 20 (TBS-T) for 1 h at room temperature and blocked membranes were briefly washed with TBS-T. Membranes were probed with mAb PSG2 at 0.05 µg/mL in TBS-T for 1 h at room temperature and then washed (5 times, 5 min) with TBS-T. Goat anti-human IgG (Fc-specific) peroxidase (Sigma) was used at 0.02 µg/mL in TBS-T and was incubated with the membranes for 1 h at room temperature. Membranes were washed 5 times again for 5 min each with TBS-T. Bound secondary was detected with SuperSignal West Dura extended duration substrate (Pierce) using a G*:* BOX Chemi*-*XR5 fluorescence and chemiluminescence gel imaging system (Syngene) equipped with GeneSys v.1.2.5.0 software.

### Quantitative PCR

Total RNA from submandibular glands was isolated using TRIzol (Invitrogen) and the RNeasy® kit (Qiagen) according to the manufacturer’s instructions. cDNA was prepared using the iScript cDNA synthesis kit (Bio-Rad), and real-time quantitative PCR was performed using SYBR Green PCR master mix (Applied Biosystems) and the CFX96 Real-Time PCR Detection System (BioRad). The following qPCR primers were used: *Tpst1* (5’-CGG GTG TCA CAG ATG AAG TG -3’ and 5’-GTC AAG GAT TTC AGG GCA AA-3’); *Tpst2* (5’-TGC CCG TGT ACT ATG AGC AG-3’ and 5’-GCT CGA TCT TGG ACA AGG AG-3’); *Gapdh* (5’-TCA ACG GCA CAG TCA AGG-3’ and 5’-ACT CCA CGA CAT ACT CAG C-3’); and *β-actin* (5’-TGT TAC CAA CTG GGA CGA CA-3’ and 5’-GGG GTG TTG AAG GTC TCA AA-3’). The relative normalized expression was determined using the comparative threshold cycle (C_T_) method and the β-actin and GAPDH housekeeping genes as internal controls.

## Results

### Salivary gland structure in wild type, *Tpst1-/-*, and *Tpst2-/-* mice

We previously reported that *Tpst2-/-* mice on a 129S6 genetic background have mild-moderate primary hypothyroidism, whereas *Tpst1-/-* mice are euthyroid [18]. While using MRI to assess the size of the thyroid gland in *Tpst2-/-* mice we noticed that the salivary glands appeared somewhat smaller than in wild type mice. This finding prompted a detailed comparative analysis of salivary gland structure and function in wild type, *Tpst1-/-*, and *Tpst2-/-* mice.

We first performed MRI scans of the salivary glands of groups of 12-week old female wild type, *Tpst1-/-*, and *Tpst2-/-* mice (n = 3) as described in Methods. We observed that combined volume of right and left sublingual and submandibular glands in *Tpst2-/-* mice (3.26 ± 0.19 µL/g body weight) was significantly smaller than wild type (4.81 ± 0.11 µL/g body weight, *p* = 0.002) and *Tpst1-/-* glands (4.55 ± 0.41 µL/g body weight, *p* = 0.044), respectively (Figure **1**). However, the volumes of wild type and *Tpst1-/-* glands were not statistically different (*p* = 0.571).

**Figure 2:**
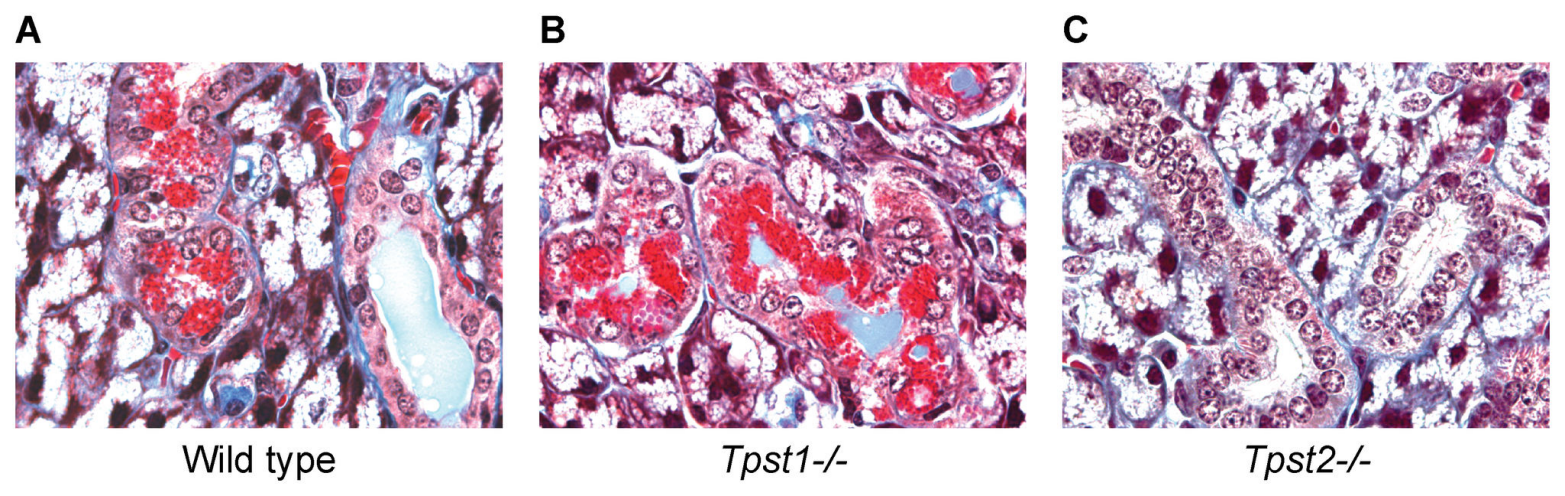
Submandibular gland histology. Submandibular glands from wild type (A) and *Tpst1-/-* (B), and *Tpst2-/-* (C) females were embedded for histology and 5 µm sections were stained with Masson’s trichrome. Images are representative of the 3 animals from the groups analysed in Figure 1. 100 x Objective.

We next performed a histological analysis of the salivary glands from the same three female wild type, *Tpst1-/-*, and *Tpst2-/-* mice. Tissues were processed for histology and 5 µm sections were stained with Masson’s trichrome (Figure **2**). Within each genotype, gland types included the sublingual gland composed predominately of mucus cells, the parotid gland predominately serous cells and, the sexually dimorphic submandibular gland composed of mixed mucus and serous cells and replete with granulated convoluted tubules (GCTs). No obvious differences in the parotid or sublingual glands were evident between the genotypes (not shown). In the submandibular glands of wild type and *Tpst1-/-* mice, GCT cells contained abundant exocrine-type cytoplasmic secretory granules that were prominently visible in the Masson’s stain. However, in *Tpst2-/-* submandibular glands, GCT cells were almost completely devoid of Masson’s positive secretory granules. The overall prevalence of the granulated tubules in the section was consistent with the female sex of the mice.

### Salivary gland function in wild type, *Tpst1-/-*, and *Tpst2-/-* mice

**Figure 3:**
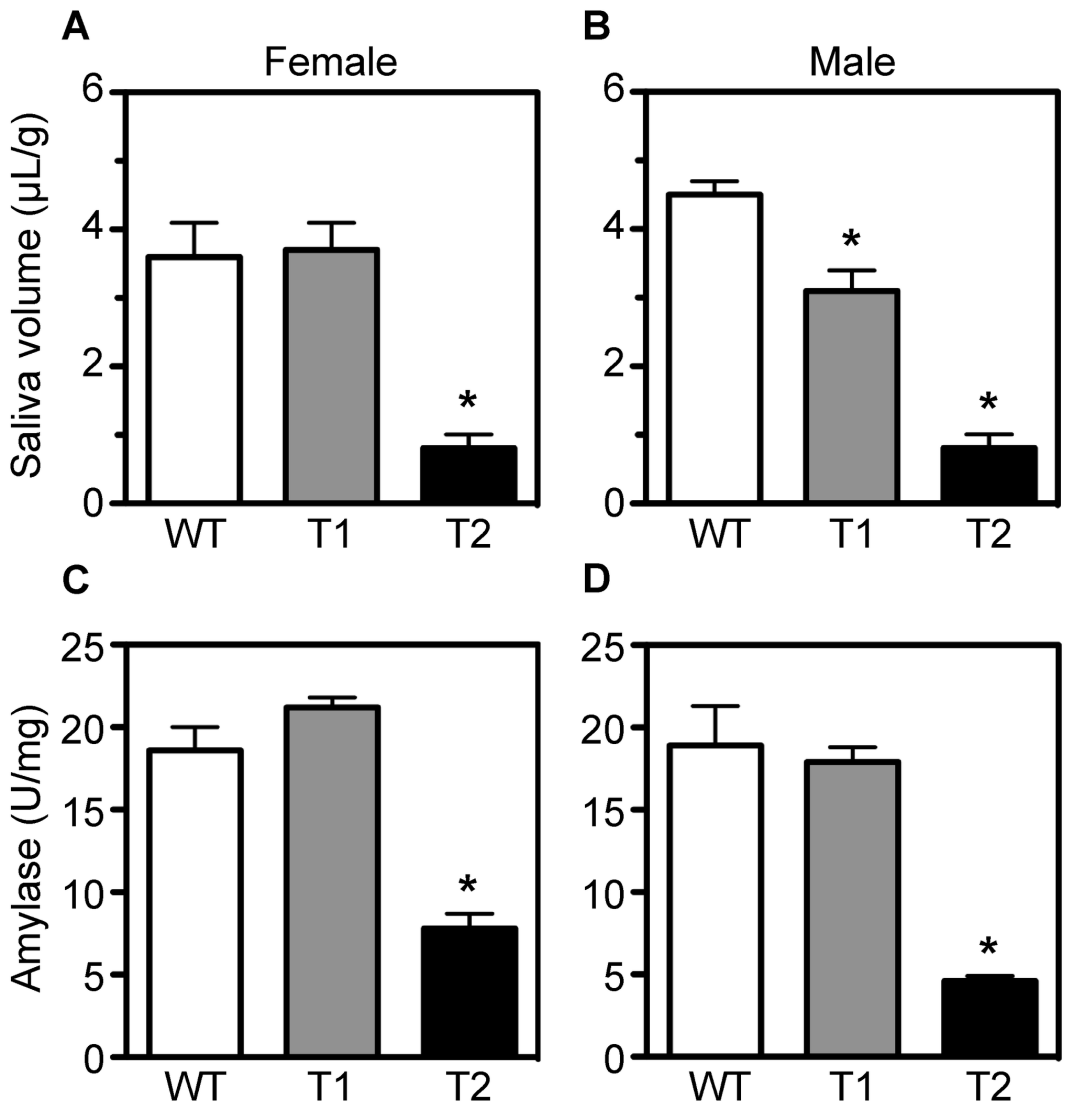
Salivary gland function in wild type, *Tpst1-/-*, and *Tpst2* -/- mice. Saliva was collected from age-matched wild type (WT), *Tpst1-/-* (T1), and *Tpst2-/-* (T2) males and females as described in Methods. Pilocarpine-induced salivary flow normalized to body weight in grams (A, B) and α-amylase activity normalized to protein concentration (C, D) were measured as described in Methods. All results are represented as the mean ± S.E.M. For wild type and *Tpst1-/-* males and females, n = 5. For *Tpst2-/-* males and female, n = 3 and 4, respectively. Statistical differences between groups were tested using unpaired, two-tailed *t*-tests with equal sample variance and an α ≤ 0.05. An asterisk indicates *p* < 0.01 compared to wild type values.

To assess salivary gland function, we measured pilocarpine-induced salivary flow and saliva composition in a second cohort of wild type, *Tpst1-/-*, and *Tpst2-/-* mice as described in Methods. For females, salivary flow and salivary α-amylase activity in *Tpst2-/-* animals was substantially lower than in wild type and *Tpst1-/-* animals. Likewise, in males, salivary flow and salivary α-amylase activity in *Tpst2-/-* animals was also lower than in wild type and *Tpst1-/-* animals (Figure **3**). For females, salivary flow and salivary α-amylase activity were the same in wild type and *Tpst1-/-* animals. For males, salivary flow was lower in *Tpst1-/-* than in wild type animals, but salivary α-amylase activity was the same. In addition, no consistent difference was discernible in the general profile of proteins in saliva between wild type, *Tpst1-/-*, and *Tpst2-/-* mice as assessed by SDS-PAGE (Figure **S1A**). Taken together, these data demonstrate that *Tpst2-/-* mice, but not *Tpst1-/-* mice, have hypofunctional salivary glands characterized by low pilocarpine-induced salivary flow and low salivary α-amylase activity.

### TPST expression in submandibular gland

**Figure 4:**
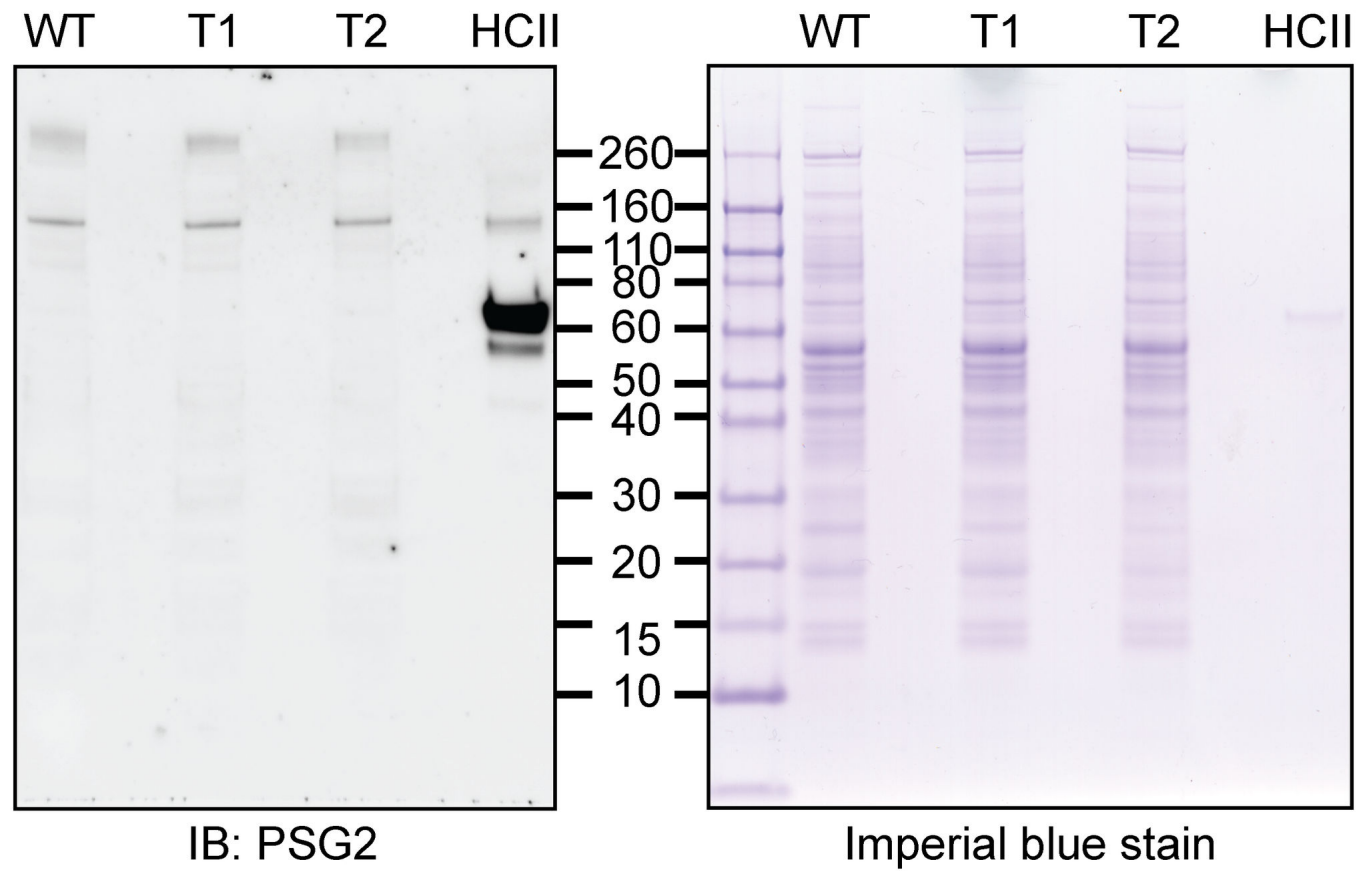
Sulfotyrosine Western blot analysis of salivary glands. Submandibular, sublingual, and parotid glands from wild type (WT), *Tpst1-/-* (T1), and *Tpst2-/-* (T2) mice were collected, homogenized and post-nuclear supernatant were prepared as described in Methods. Non-reduced proteins (10 µg per lane) were resolved in 4-12% Bis-Tris polyacrylamide gels. Proteins were either transferred onto nitrocellulose membranes and probed with PSG2 followed by HRP conjugated secondary antibody (A) or detected with Imperial protein stain (ThermoScientific) (B). Human heparin cofactor II (HCII), a known tyrosine-sulfated protein, served as a positive control.

We next addressed the question whether there are different levels of tyrosine sulfation on proteins in salivary glands in wild type, *Tpst1-/-*, and *Tpst2-/-* mice. We conducted a Western blot analysis of salivary gland extracts using the PSG2 anti-sulfotyrosine mAb. A number of tyrosine-sulfated proteins were detected consistent with the previous detection of TPST activity in salivary glands [9] (Figure **4**). However, there were no discernible differences between homogenates from wild type, *Tpst1-/-*, and *Tpst2-/-* animals. A similar analysis showed that no tyrosine-sulfated proteins were detected in pilocarpine-induced saliva from male or female wild type, *Tpst1-/-*, and *Tpst2-/-* animals (Figure **S1B**).

To assess the relative expression of TPST-1 and TPST-2 in submandibular glands, qPCR analyses were performed (Figure **S2**). We observed that *Tpst1* and *Tpst2* transcripts are present in the submandibular gland of wild type males and females (not shown). In both sexes, we detected *Tpst1* transcripts in the *Tpst2-/-* gland and *Tpst2* transcripts gland in the *Tpst1-/-* gland consistent with the detection of tyrosine-sulfated proteins in both *Tpst1-/-* and *Tpst2-/-* mice. Finally, we observed that the level of *Tpst2* transcripts in the *Tpst1-/-* gland is equivalent to wild type levels. However, *Tpst1* transcripts were ^≈^ 50% higher in the *Tpst2-/-* gland compared to wild type. Taken together, these data suggest that the salivary gland hypofunction in *Tpst2-/-* mice is not due to defective sulfation of proteins in saliva or the salivary glands proper, but instead suggests that the salivary gland hypofunction is due to factor(s) extrinsic to the salivary glands.

### Thyroid hormone rescue

**Figure 5:**
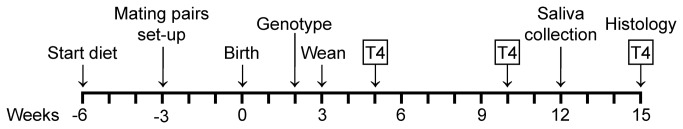
Schematic diagram of thyroid hormone rescue protocol and timing of experimental endpoints. Sexually mature *Tpst2+/-* males and females were conditioned to control chow or chow supplemented with thyroid powder for 3 weeks. Mating pairs were then established and continued on the control or experimental diet throughout pregnancy. Weanlings were then placed on the same diet until they were sacrificed at 15 weeks of age. All animals were weighed weekly and serum total T4 levels were determined at 5, 10, and 15 weeks of age. Saliva was collected at 12 weeks of age and animals were sacrificed at 15 weeks of age for histological analysis. Groups of 10-13 animals of each genotype (Tpst2+/+, *Tpst2+/-*, *Tpst2-/-*) and sex were included in the study.

As mentioned above *Tpst2-/-* mice have mild-moderate primary hypothyroidism, whereas *Tpst1-/-* mice are euthyroid [18]. We therefore asked if the functional and structural changes in the salivary gland we observe in *Tpst2-/-* mice could be rescued by thyroid hormone replacement. The rescue protocol and timing of experimental endpoints is illustrated in Figure **5**. Briefly, sexually mature *Tpst2+/-* males and females were conditioned to control chow or chow supplemented with thyroid powder for 3 weeks. Mating pairs were then established and continued on the control or experimental diet throughout pregnancy and pups were place on the same diet until they were sacrificed at 15 weeks of age. All animals were weighed weekly, serum T4 levels were determined at 5, 10, and 15 weeks of age, saliva was collected at 12 weeks of age, and animals were sacrificed at 15 weeks of age for histological analysis. Groups of at least 10 animals of each genotype (Tpst2+/+, *Tpst2+/-*, *Tpst2-/-*) and sex were included in the study. No significant differences were observed between Tpst2+/+ and *Tpst2+/-* animals for any of the experimental endpoints. Therefore, only data for Tpst2+/+ and *Tpst2-/-* are presented.

**Figure 6:**
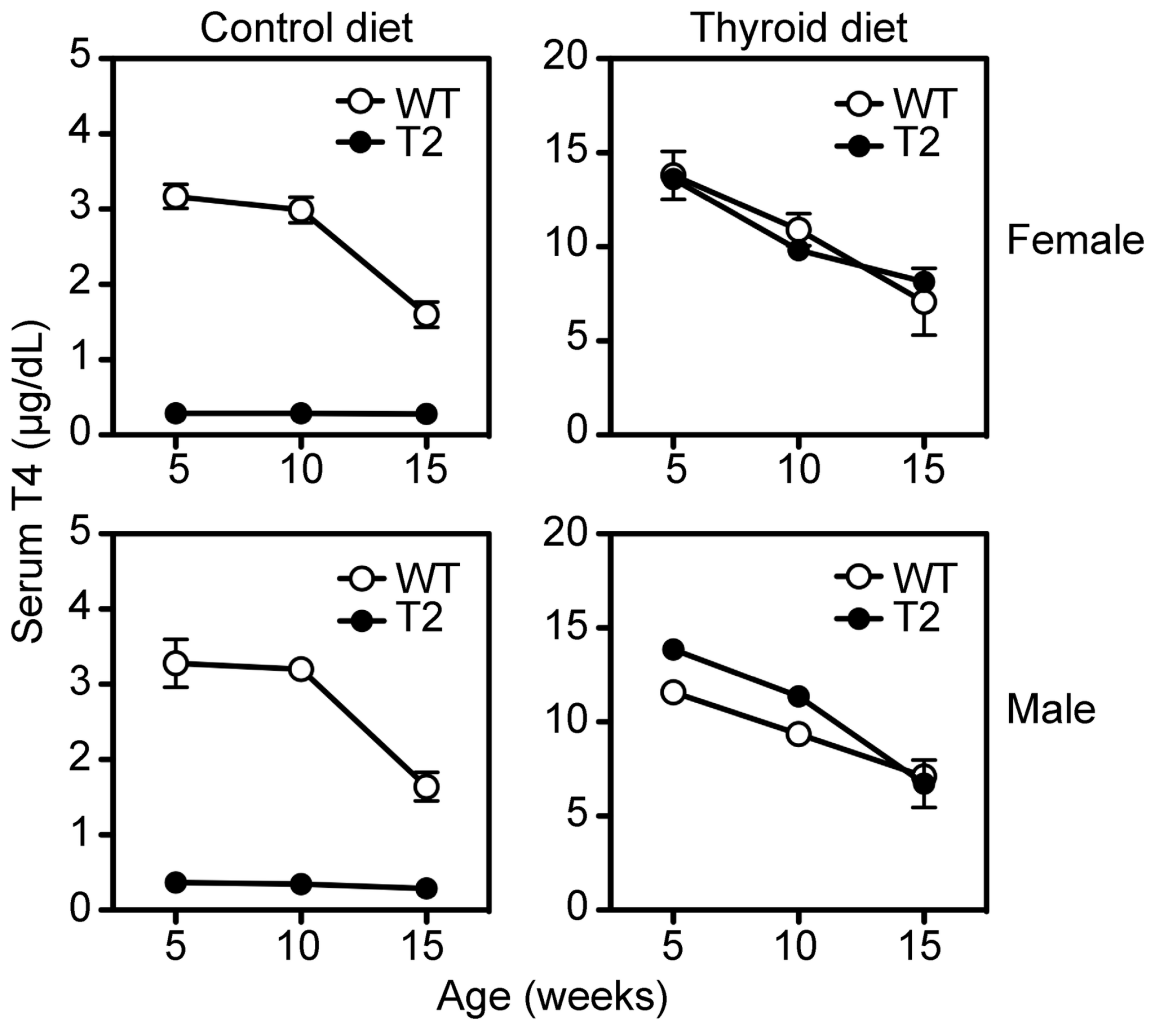
Serum T4 levels. Serum total T4 levels were determined at 5, 10, and 15 weeks of age for 4 mice randomly selected from the 10-13 mice in each experimental group. WT = wild type. T2 = *Tpst2-/-*. Results are expressed as mean ± S.E.M.

The first endpoint of the rescue experiment was serum total T4 levels at 5, 10, and 15 weeks of age to document the adequacy of thyroid hormone replacement. On the control diet, the serum T4 levels in wild type females and males are the same at 5 and 10 weeks of age (Figure **6**, left panels), but drop by ^≈^ 50% between 10 and 15 weeks of age. In contrast, we observe very low serum T4 levels in *Tpst2-/-* mice at all ages tested and irrespective of sex, consistent with our previously reported results in adult *Tpst2-/-* males [18]. On the experimental diet, T4 levels are indistinguishable at 5, 10, and 15 weeks of age irrespective of genotype or sex, but are generally 3-4 times higher than animals on the control diet (Figure **6**, rights panels). Again, we observe a gradual drop in serum T4 levels as a function of age in both males and females. These data demonstrate the adequacy of thyroid hormone replacement.

**Figure 7:**
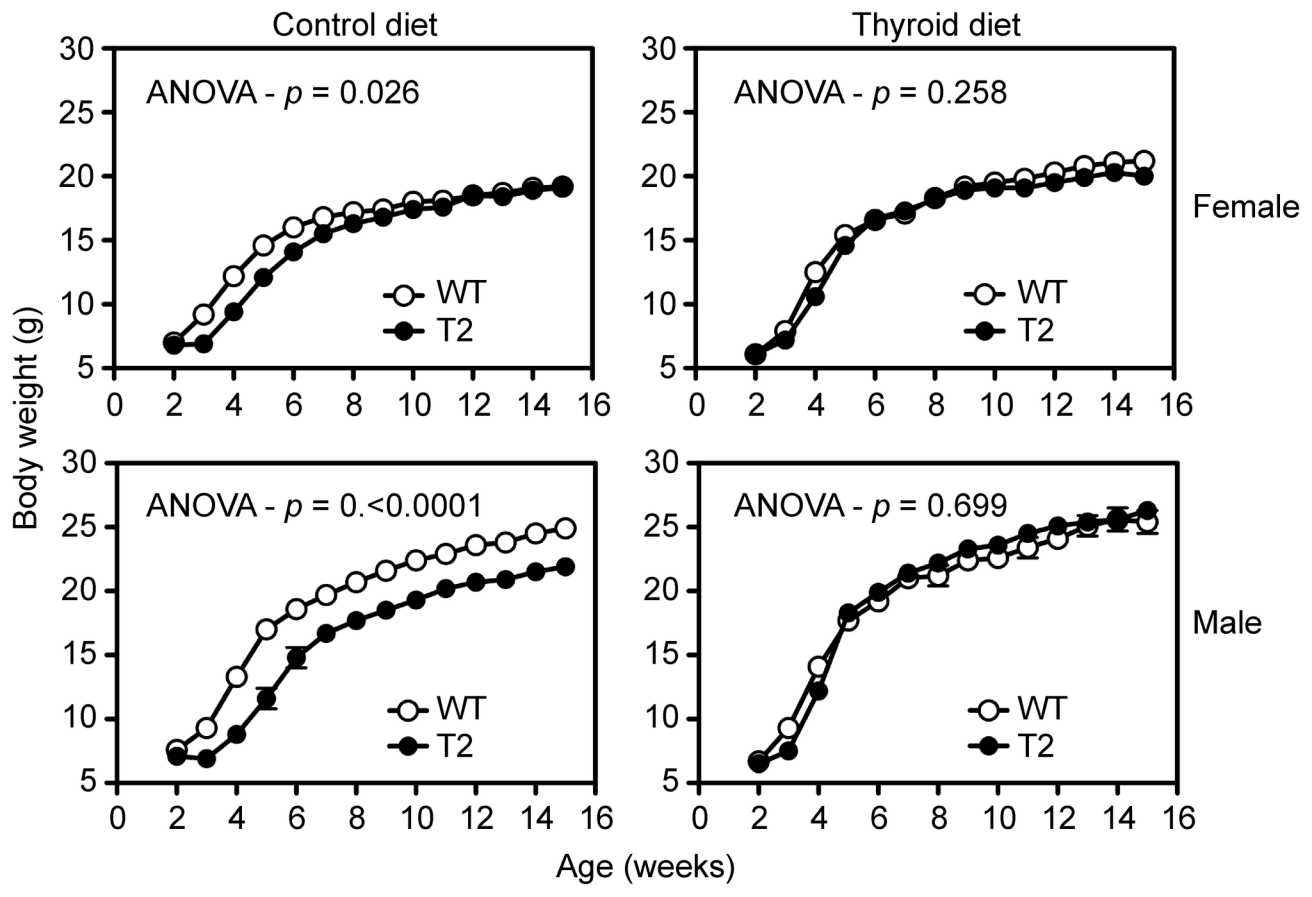
Body weights. Body weight were measured weekly for all mice in each experimental group (n = 10-13). WT = wild type. T2 = *Tpst2-/-*. Results are expressed as mean ± S.E.M. Differences between groups was assessed with a two-way repeated-measures ANOVA using Prism 6 software. Statistical differences at each age were tested *post-hoc* using unpaired, two-tailed *t*-tests with equal sample variance and an α ≤ 0.05.

The second endpoint of the rescue experiment was body weight as a function of age. On the control diet, the body weights of *Tpst2-/-* females were significantly lower than those of wild type littermates from 3 to 7 weeks of age (Figure **7**, top left). However, on the experimental diet the body weights of wild type and *Tpst2-/-* females were indistinguishable at all ages (Figure **7**, top right). In addition, body weights of wild type females on the control and experimental diets were indistinguishable up to 8 weeks of age after which the weights of the animals on the experimental diet exceeded those on the control diet (Figure **S3**, top left). Similarly, the body weights of *Tpst2-/-* males were significantly lower than those of wild type littermates from 3 to 15 weeks of age (Figure **7**, bottom left). However, on the experimental diet the body weights of wild type and *Tpst2-/-* males were indistinguishable (Figure **7**, bottom right). In addition, the body weights of wild type males on the control and experimental diet were indistinguishable (Figure **S3**, bottom left). These data demonstrate that the body weights of *Tpst2-/-* males and females are lower than wild type littermates consistent with previous results [12] and that this phenotype is completely rescued by thyroid hormone replacement.

**Figure 8:**
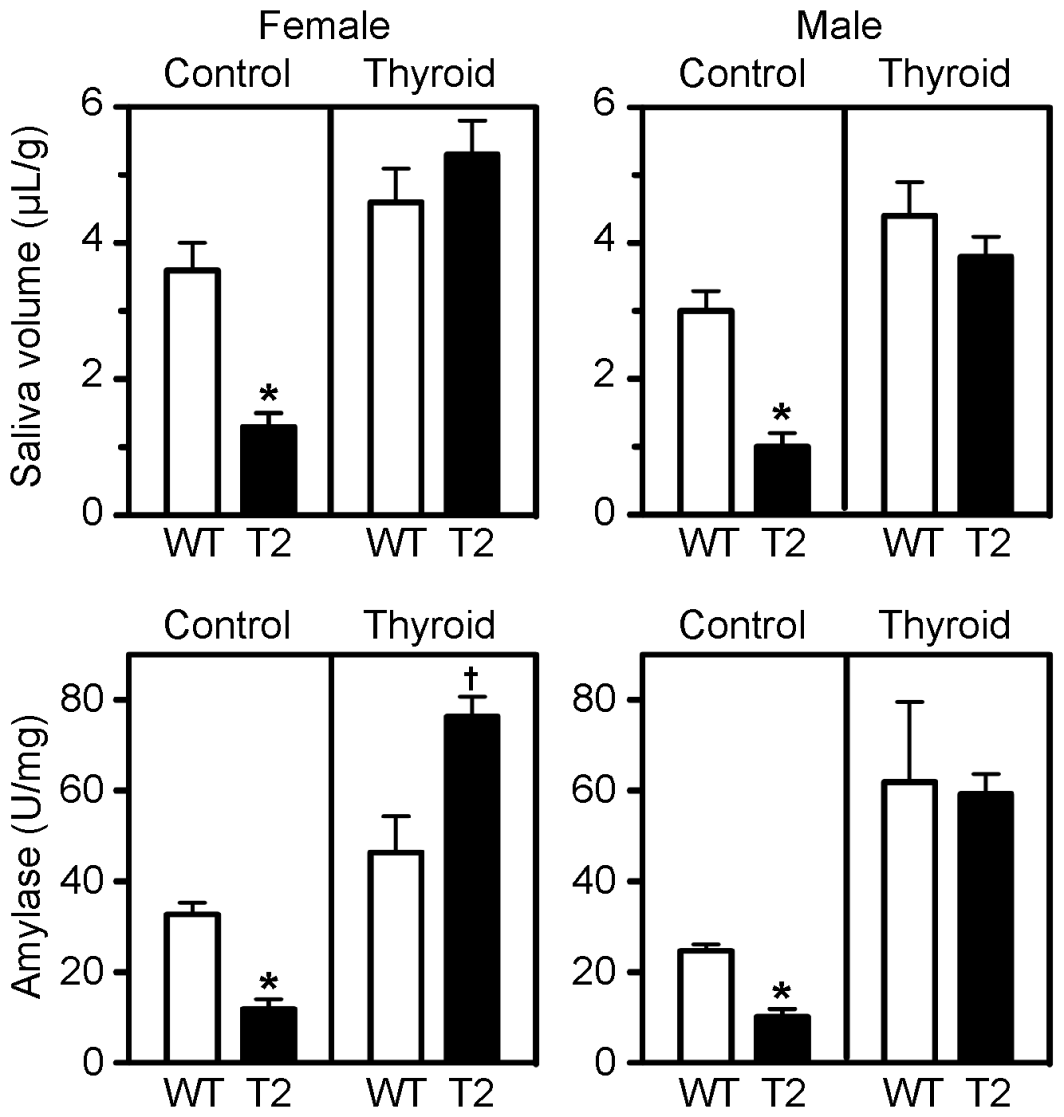
Salivary gland function. Saliva was collected at 12 weeks of age from the two experimental groups as described in Methods. Pilocarpine-induced salivary flow normalized to body weight in grams (A, B) and α-amylase activity normalized to protein concentration (C, D) were measured as described in Methods. Salivary flow for all mice in each experimental group, whereas α-amylase activity was measured in 4 mice randomly selected from the 10-13 mice in each experimental group. WT = wild type. T2 = *Tpst2-/-*. Results are expressed as mean ± S.E.M. Statistical differences between groups were tested using unpaired, two-tailed *t*-tests with equal sample variance and an α ≤ 0.05. An asterisk indicates *p* < 0.01 compared to wild type values. A lancet indicates *p* < 0.02 compared to wild type values.

The third endpoint of the experiment was pilocarpine-induced salivary flow and saliva composition at 12 weeks of age. For females on the control diet, both salivary flow (*p* = 0.004) and salivary α-amylase activity (*p* < 0.001) in *Tpst2-/-* animals were significantly lower than in wild type littermates (Figure **8**). This same overall pattern was observed for males. In this cohort we observed that α-amylase activity (*p* = 0.034) was significantly higher in females than in males. On the experimental diet, we observed no statistically significant differences between *Tpst2-/-* and wild type littermates with respect to salivary flow, or salivary α-amylase activity, except that α-amylase activity was higher in *Tpst2-/-* females on the experimental diet than in wild type controls (*p*= 0.016). Taken together, these data demonstrate that thyroid hormone replacement reverses salivary gland hypofunction in *Tpst2-/-* females and males.

**Figure 9:**
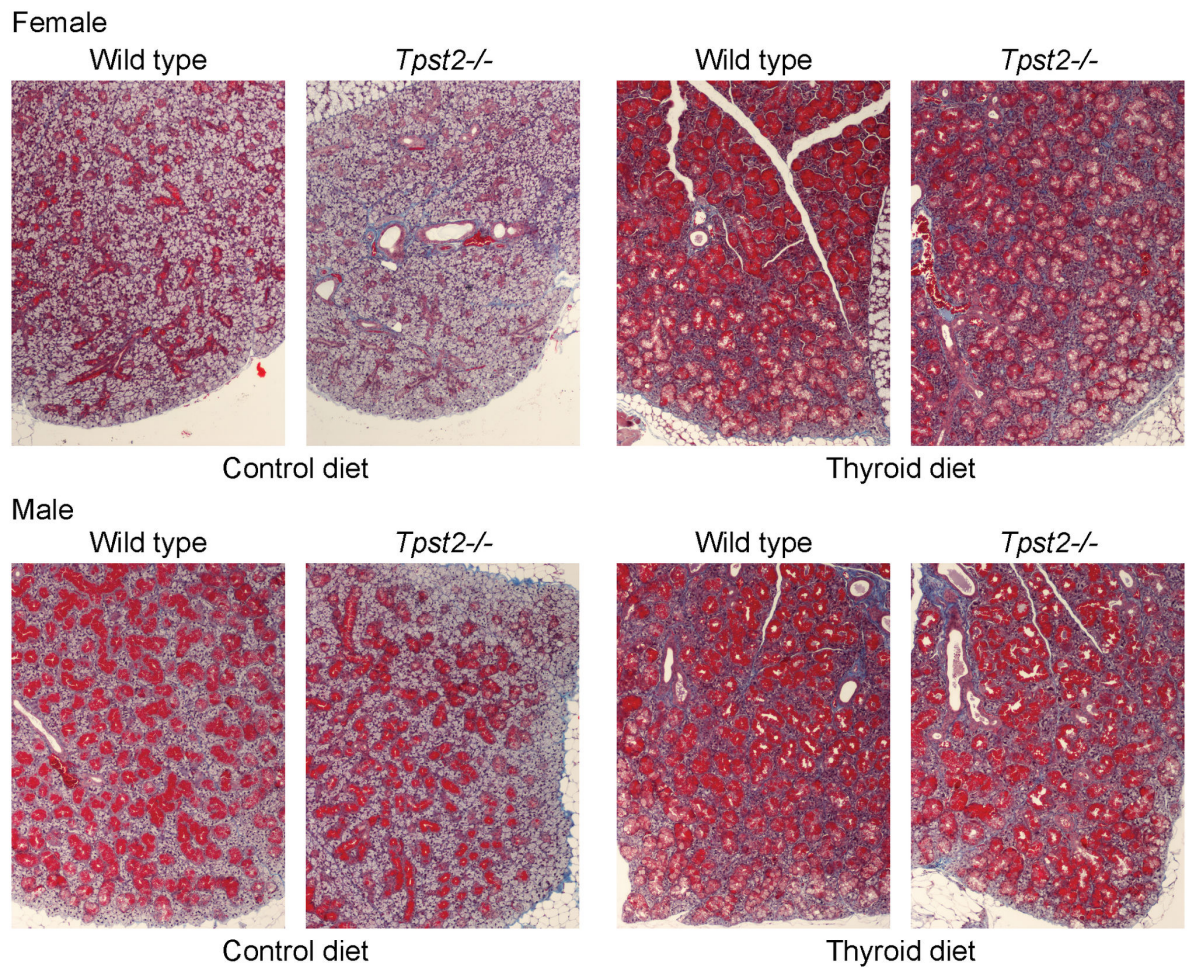
Submandibular gland histology. At 15 weeks of age submandibular glands from female (top panels) or males (bottom panels) were harvested and then fixed, wax embedded, sectioned, and stained with Masson’s trichrome as described in Methods. Images are representative of analyses of the 4 mice randomly selected from the >10 mice in each experimental group in Figure 7. 100 x Objective.

The final endpoint of the experiment was to conduct a detailed histological examination. Four of the 10 animals of each sex, genotype, and diet group were randomly selected for these studies (32 animals total). On the control diet, submandibular glands from wild type males showed clear sexual dimorphism compared to wild type females on the same diet (Figure **9**, left panels and Figure **S4**). In the male submandibular gland, ^≈^ 80% of the structure consisted of GCT cells containing abundant distinct Masson’s positive exocrine-type cytoplasmic secretory granules. However, in the female gland, GCT cells contributed to ^≈^ 40-50% of the gland structure and showed less prominent cytoplasmic granulation.

Sexual dimorphism was also evident between male and female *Tpst2-/-* mice on the control diet. In the male submandibular glands, GCT cells occupied ^≈^ 60-70% of the glandular structure and cytoplasmic granules were strongly Masson’s positive, whereas in female glands 30-40% of the structure was made up of granulated tubules. However, Masson’s positive granulation was dramatically reduced in the female.

In general, all mice on the thyroid hormone supplemented diet demonstrated an increase in the proportion of GCTs comprising the structure of the submandibular glands and an increase in the granule size variation within the GCT cells. Many cells contained large Masson’s positive globular granules (Figure **9**, right panels and Figure **S5**). In males, the wild type and *Tpst2-/-* animals showed a microscopically discernible increase in GCT proportion of glandular structure. In females, wild type mice showed a ^≈^ 30% increase in the GCT proportion, while *Tpst2-/-* mice showed a ^≈^ 40-50% increase, estimated microscopically. Furthermore, in wild type animals, male GCTs were characterized by an increase in coarseness, a larger variation in granule size and the presence of globular and amphophilic inclusions in GCT cells. In females, GCT cells were characterized by an increase in granule coarseness and diversity of granule size, as well as some evidence of globular inclusions.

## Discussion

In a previous study we showed that *Tpst2-/-* mice had mild-moderate primary hypothyroidism, whereas *Tpst1-/-* mice were euthyroid. During our analysis of *Tpst2-/-* mice we used MRI to image the thyroid gland and we noticed that the salivary glands appeared smaller than in wild type mice. In this study we confirmed that salivary glands were ^≈^ 30% smaller in *Tpst2-/-* females compared to wild type or *Tpst1-/-* females using quantitative MRI imaging (Figure **1**). We further documented the granular convoluted tubules in *Tpst2-/-* submandibular glands were less prominent and were almost completely devoid of exocrine secretory granules compared to glands from wild type or *Tpst1-/-* females (Figure **2**). In addition, pilocarpine–induced salivary flow and salivary α-amylase activity in *Tpst2-/-* animals of both sexes was substantially lower than in wild type and *Tpst1-/-* animals (Figure **3**). Thus, we conclude that *Tpst2-/-*, but not *Tpst1-/-* mice, have salivary gland hypofunction.

We next sought some insight as to the mechanism for the salivary gland hypofunction. We first used anti-sulfotyrosine Western blotting of salivary glands and saliva to indirectly assess the level of protein-tyrosine sulfation in the glands. We observed identical protein sulfation patterns in the parotid, sublingual, and submandibular gland extracts (Figure **4**) and in pilocarpine–induced saliva sample from wild type, *Tpst1-/-*, and *Tpst2-/-* animals (Figure **S1**). This is a common finding in many other tissues ( [22] and unpublished observations) that is likely due to the fact that all tissues and cells express both TPST-1 and TPST-2 transcripts and that there is an extensive overlap in the macromolecular substrate specificities of the two enzymes. Only occasionally have we observed major differences in the protein sulfation patterns in tissues, one example being the epididymis [20,23]. The identity of the salivary proteins we detected by anti-sulfotyrosine Western blotting are not known and no tyrosine-sulfated salivary proteins have been identified in the mouse. Two human salivary proteins, statherin and histatin-1, have been shown to be tyrosine-sulfated, but orthologs are not present in the mouse genome [24,25]. Nevertheless, our data suggest that the salivary gland hypofunction is due to factor(s) extrinsic to the salivary glands.

Studies published in the 1950s showed that induction of thyroid gland dysfunction in the rat by propylthiouracil or ^131^Iodine administration resulted in reduced salivary flow accompanied by histological changes in the salivary glands [26–29] indicating that the thyroid hormone has a trophic effect on the salivary gland. These data prompted us to assess the impact of thyroid hormone supplementation on the salivary gland hypofunction. We found that all indicators of hypothyroidism (serum T4 and body weight) and salivary gland hypofunction (pilocarpine-induced salivary flow, α-amylase activity, and histological changes) were restored to normal or near normal by thyroid hormone supplementation. These studies conclusively demonstrate that salivary gland hypofunction in *Tpst2-/-* mice is due solely to primary hypothyroidism.

In our studies, we noted sexually dimorphic characteristics in the submandibular gland that are consistent with studies published in the 1940s [30,31]. Salivary α-amylase activity (Figure **8**) was significantly higher in females than in males. In addition, sexual dimorphism was also apparent histologically (Figure **9** and Figures **S4** & **S5**). A much higher proportion of male submandibular gland consisted of GCT cells with much more abundant Masson’s positive cytoplasmic secretory granules compared to the female both on the control and the thyroid supplemented diet.

As to the mechanism(s) for the hypothyroidism, it is likely that this is due to undersulfation of the thyroid-simulating hormone receptor (TSH-R), a member of a subfamily of the G protein-coupled receptor superfamily that includes the luteinizing hormone and follicle-stimulating hormone receptors. Previous studies have shown that a recombinant glycosyl-phosphatidylinositol*-*linked form of the human TSH-R ectodomain was sulfated when expressed in COS-7 cells [32,33]. The site of sulfation was localized to the hinge region at two sites within a YDY motif that is conserved in the mouse and many other species. This motif is located close to the first transmembrane helix in the full-length receptor. Sulfation of the first tyrosine (Y^385^) within the YDY motif was required for high affinity binding of TSH and receptor activation.

In 2007, it was reported that a *Tpst2* gene mutation (C798G) causes autosomal recessive primary hypothyroidism in the growth-retarded *grt*/*grt* mouse [19]. This mutation results in a H266Q substitution in TPST-2. Evidence was presented that the *grt* mutation leads to a loss of TPST-2 activity. In addition, it was shown that TPST-2, but not TPST-1 or the H266Q TPST-2 mutant, can sulfate a synthetic peptide modeled on the sulfation site in the TSH-R, suggesting that TPST-2 is required for TSH-R sulfation *in vivo*. However, it remains to be determined if the native TSH-R is undersulfated in *Tpst2-/-* mice.

It is interesting that disrupting the *Tpst2* gene results in a much more robust phenotype(s) than disrupting the *Tpst1* gene. In the *Tpst2* knockout, we find both male sterility and primary hypothyroidism, but only very subtle changes in the *Tpst1-/-* knockout. The basis for this may in part be related to differences in the macromolecular substrate specificity of the two enzymes as suggested by the data with the *grt* mutation. However, difference in the relative abundance of the two isoenzymes in different tissues and cell types may also contribute. We and others have reported differences in the relative abundance of *Tpst1* and *Tpst2* transcripts in mouse and human tissues [11,12,34]. However, this provides limited insight into the relative abundance or physiological importance of TPST-1 and TPST-2 proteins in any given tissue or cell. We have been frustrated in our efforts to address this question over the years by our inability to detect and quantitate native TPSTs in tissues. Despite the fact that we have successfully developed isoenzyme specific polyclonal and monoclonal antibodies, these have proven to be unable to detect their respective targets either in Western blots and ELISAs of tissue extracts, or by immunofluorescence microscopy of tissues. This may be because our reagents lack sufficient affinity and/or avidity and/or because the enzymes are very low in abundance.

## Supporting Information

Figure S1 **Protein and anti-sulfotyrosine Western blot analysis of saliva.** Pilocarpine-induced saliva was collected from age-matched male and female mice as described in Methods. (A) Saliva from wild type (WT), *Tpst1-/-* (T1), and *Tpst2-/-* (T2) mice (10 µg of protein) were resolved in 4-12% Bis-Tris polyacrylamide gels under reducing conditions and subjected to Silver staining (ThermoScientific). The analysis shown is representative of 3 independent experiments. (B) Saliva from wild type male and female mice (10 and 80 µg of protein) were resolved in 4-12% Bis-Tris polyacrylamide gels under reducing or non-reducing conditions, transferred onto nitrocellulose membranes, and probed with PSG2 followed by HRP conjugated secondary antibody. Human heparin cofactor II (HCII), a known tyrosine-sulfated protein, served as a positive control.
(PDF)

Figure S2 **TPST expression in submandibular gland.** Total RNA from submandibular glands was isolated, cDNA was prepared, and real-time quantitative PCR using primers specific for *Tpst1*, *Tpst2*, *Gapdh*, and *β-actin* were performed as described in Methods. WT = wild type. T1 = *Tpst1-/-*. T2 = *Tpst2-/-*. The relative normalized expression was determined using the comparative threshold cycle (C_T_) method and the β-actin and GAPDH housekeeping genes as internal controls. Results are expressed as mean ± S.E.M, n = 4. Differences between groups was assessed with a two-way ANOVA using Prism 6 software. Statistical differences between genotypes were tested post-hoc using a Bonferroni multiple comparisons test and an α ≤ 0.05. An asterisk indicates p < 10^-4^ compared to wild type values.
(PDF)

Figure S3 **Body weights.** Body weight were measured weekly for all mice in each experimental group (n = 10-13). WT = wild type. T2 = *Tpst2-/-*. Results are expressed as mean ± S.E.M. Differences between groups was assessed with a two-way repeated-measures ANOVA using Prism 6 software. Statistical differences at each age were tested *post-hoc* using an unpaired, two-tailed *t*-test with equal sample variance and an α ≤ 0.05.
(PDF)

Figure S4 **Submandibular gland histology on control diet.** At 15 weeks of age submandibular glands from female (top panels) or males (bottom panels) were harvested and then resin embedded, sectioned (0.5 µm) and stained with toluidine blue as described in Methods. Images are representative of analyses of the 4 mice randomly selected from the >10 mice in each experimental group in Figure 7. 63 x Objective.
(PDF)

Figure S5 **Submandibular gland histology on thyroid supplemented diet.** At 15 weeks of age submandibular glands from female (top panels) or males (bottom panels) were harvested and then resin embedded, sectioned (0.5 µm) and stained with toluidine blue as described in Methods. Images are representative of analyses of the 4 mice randomly selected from the >10 mice in each experimental group in Figure 7. 63 x Objective.
(PDF)
