# Supplementary figures and images for: Salivary Gland Hypofunction in tyrosylprotein sulfotransferase-2 Knockout Mice Is Due to Primary Hypothyroidism
Source: PLoS One. 2013 Aug 7;8(8):e71822. doi: 10.1371/journal.pone.0071822 (PMC3737198; doi:10.1371/journal.pone.0071822)

Figure S1

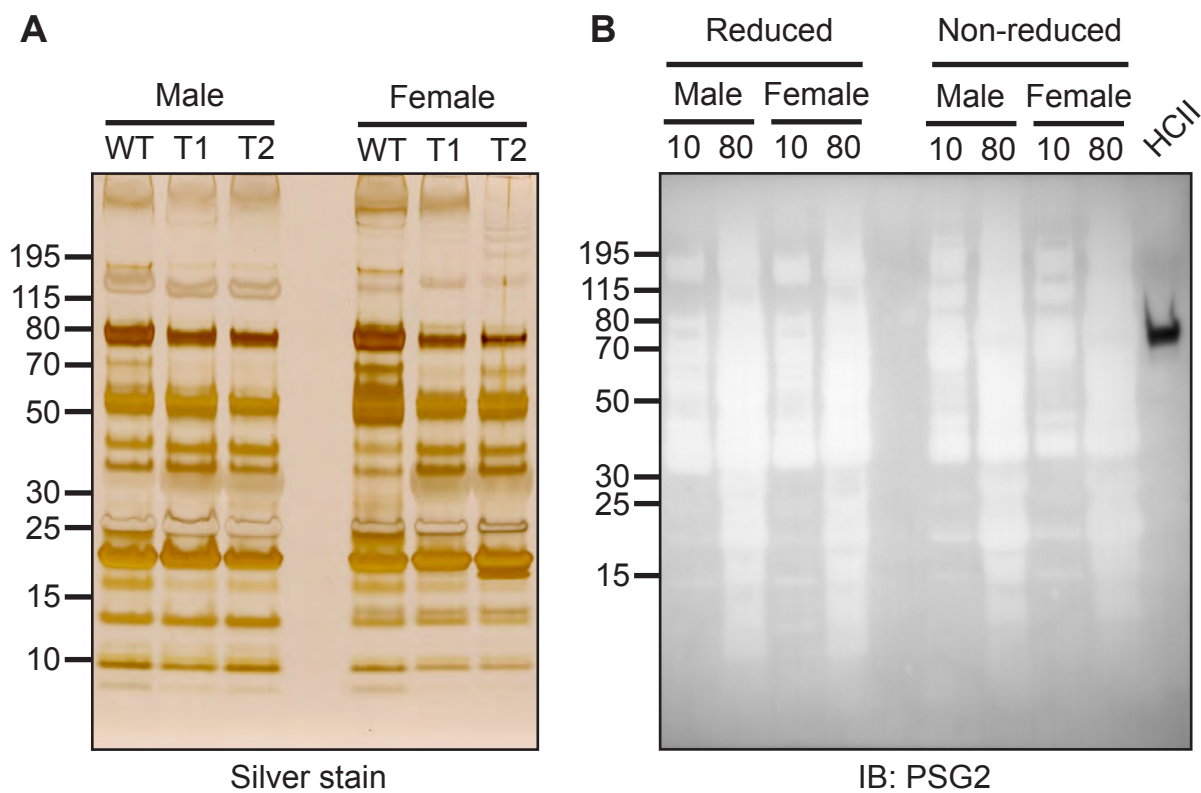

Supplement: Figure S1 — Protein and anti-sulfotyrosine Western blot analysis of saliva. Pilocarpine-induced saliva was collected from age-matched male and female mice as described in Methods. (A) Saliva from wild type (WT), Tpst1-/- (T1), and Tpst2-/- (T2) mice (10 µg of protein) were resolved in 4-12% Bis-Tris polyacrylamide gels under reducing conditions and subjected to Silver staining (ThermoScientific). The analysis shown is representative of 3 independent experiments. (B) Saliva from wild type male and female mice (10 and 80 µg of protein) were resolved in 4-12% Bis-Tris polyacrylamide gels under reducing or non-reducing conditions, transferred onto nitrocellulose membranes, and probed with PSG2 followed by HRP conjugated secondary antibody. Human heparin cofactor II (HCII), a known tyrosine-sulfated protein, served as a positive control. (PDF) [file pone.0071822.s001.pdf]

Figure S2

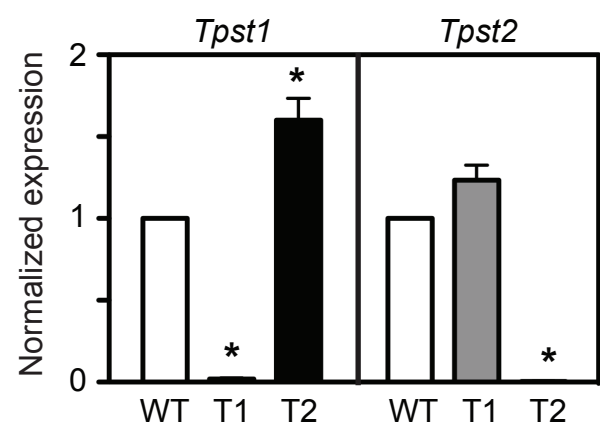

Supplement: Figure S2 — TPST expression in submandibular gland. Total RNA from submandibular glands was isolated, cDNA was prepared, and real-time quantitative PCR using primers specific for Tpst1, Tpst2, Gapdh, and β-actin were performed as described in Methods. WT = wild type. T1 = Tpst1-/-. T2 = Tpst2-/-. The relative normalized expression was determined using the comparative threshold cycle (CT) method and the β-actin and GAPDH housekeeping genes as internal controls. Results are expressed as mean ± S.E.M, n = 4. Differences between groups was assessed with a two-way ANOVA using Prism 6 software. Statistical differences between genotypes were tested post-hoc using a Bonferroni multiple comparisons test and an α ≤ 0.05. An asterisk indicates p < 10-4 compared to wild type values. (PDF) [file pone.0071822.s002.pdf]

Figure S3

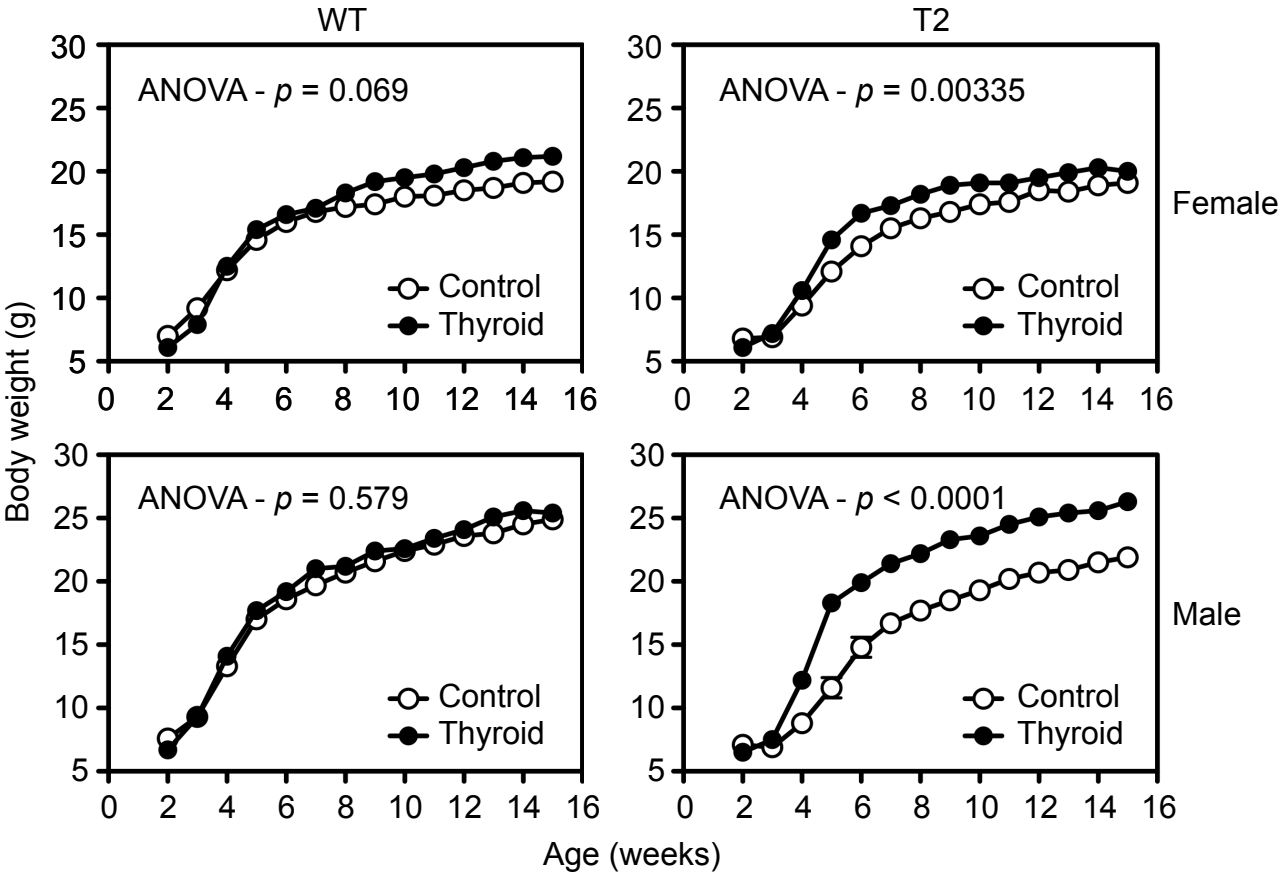

Supplement: Figure S3 — Body weights. Body weight were measured weekly for all mice in each experimental group (n = 10-13). WT = wild type. T2 = Tpst2-/-. Results are expressed as mean ± S.E.M. Differences between groups was assessed with a two-way repeated-measures ANOVA using Prism 6 software. Statistical differences at each age were tested post-hoc using an unpaired, two-tailed t-test with equal sample variance and an α ≤ 0.05. (PDF) [file pone.0071822.s003.pdf]

Figure S4

Female

Wild type

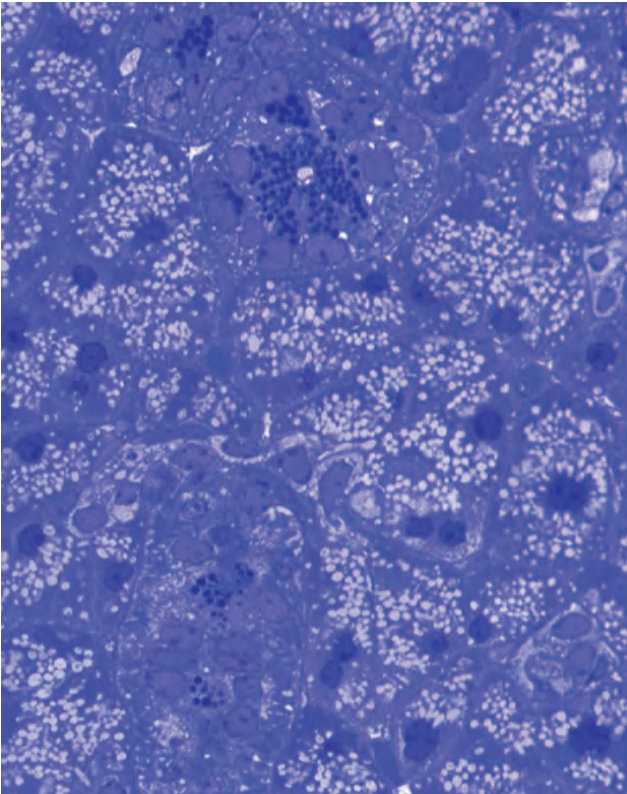

*Tpst2*<sup>-/-</sup>

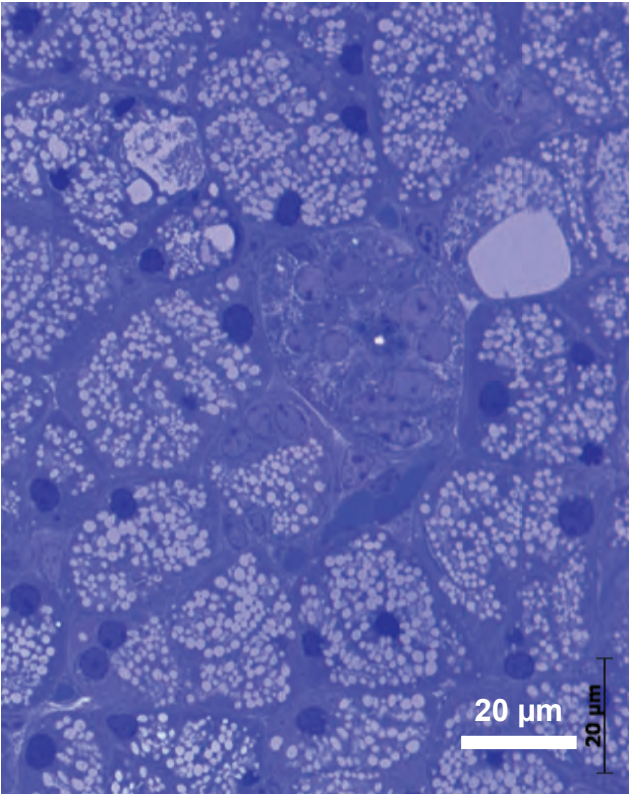

Male

Control diet

Wild type

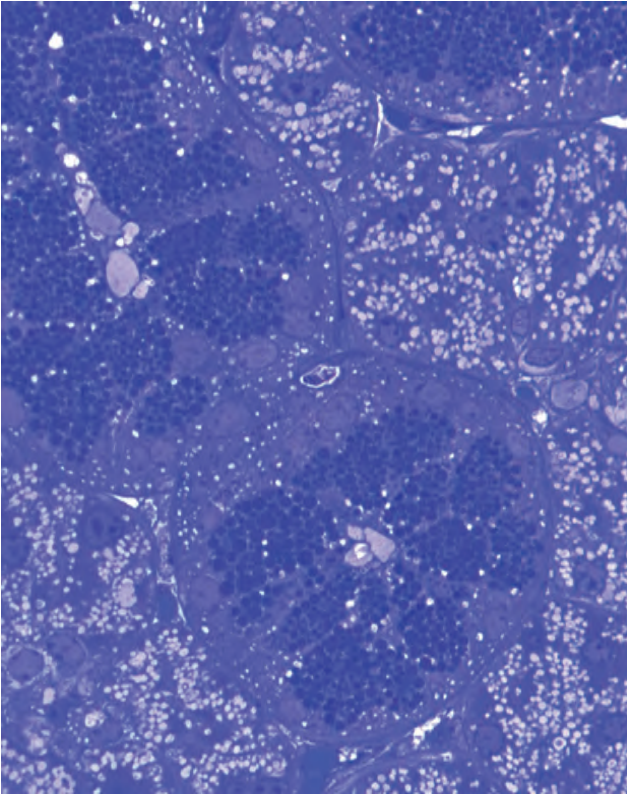

*Tpst2*<sup>-/-</sup>

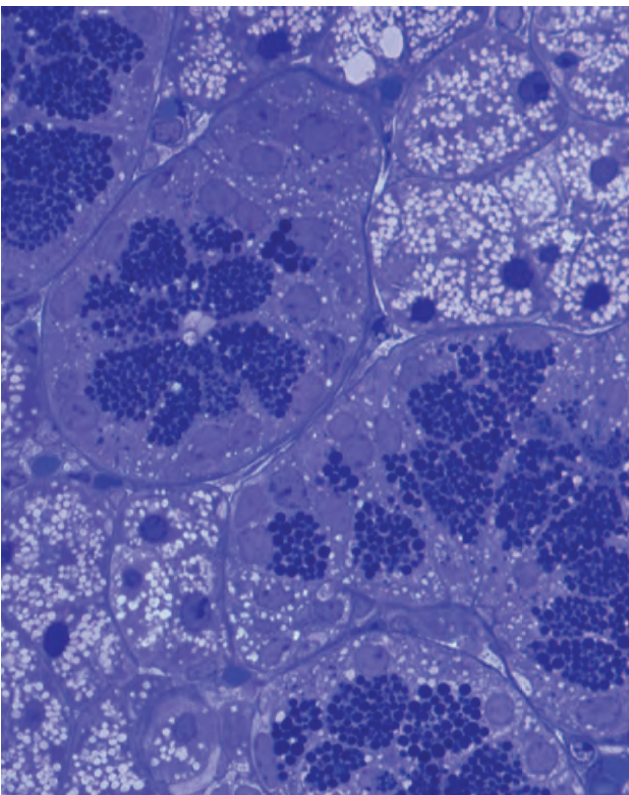

Control diet

Supplement: Figure S4 — Submandibular gland histology on control diet. At 15 weeks of age submandibular glands from female (top panels) or males (bottom panels) were harvested and then resin embedded, sectioned (0.5 µm) and stained with toluidine blue as described in Methods. Images are representative of analyses of the 4 mice randomly selected from the >10 mice in each experimental group in Figure 7. 63 x Objective. (PDF) [file pone.0071822.s004.pdf]

Figure S5

Female

Wild type

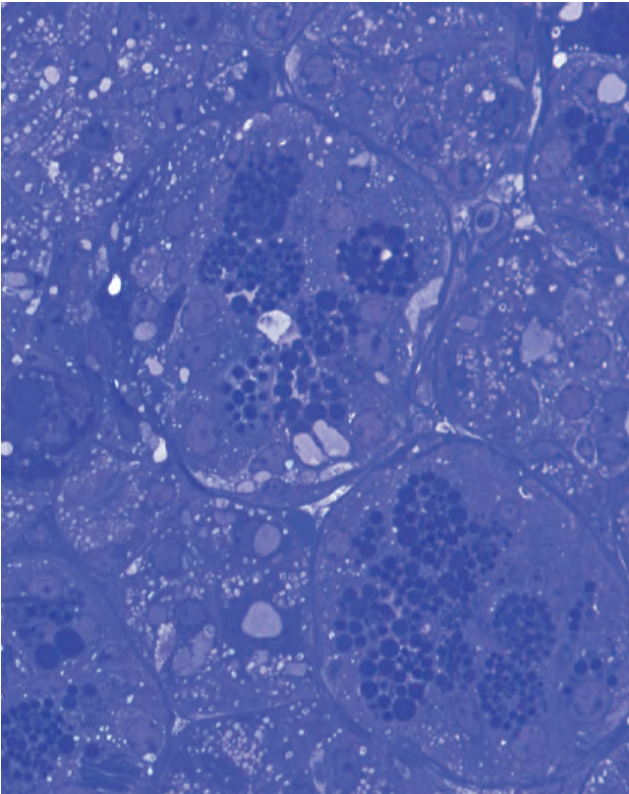

*Tpst2*<sup>-/-</sup>

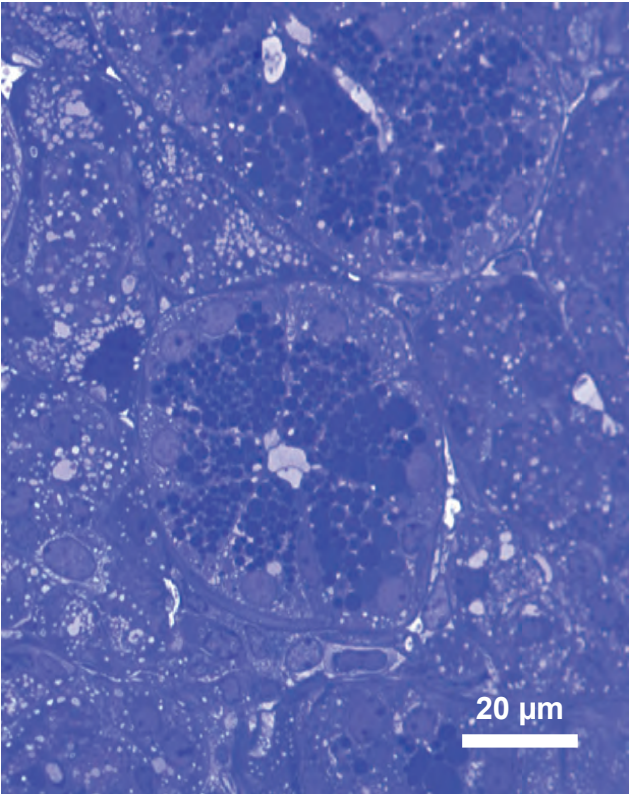

Male

Thyroid diet

Wild type

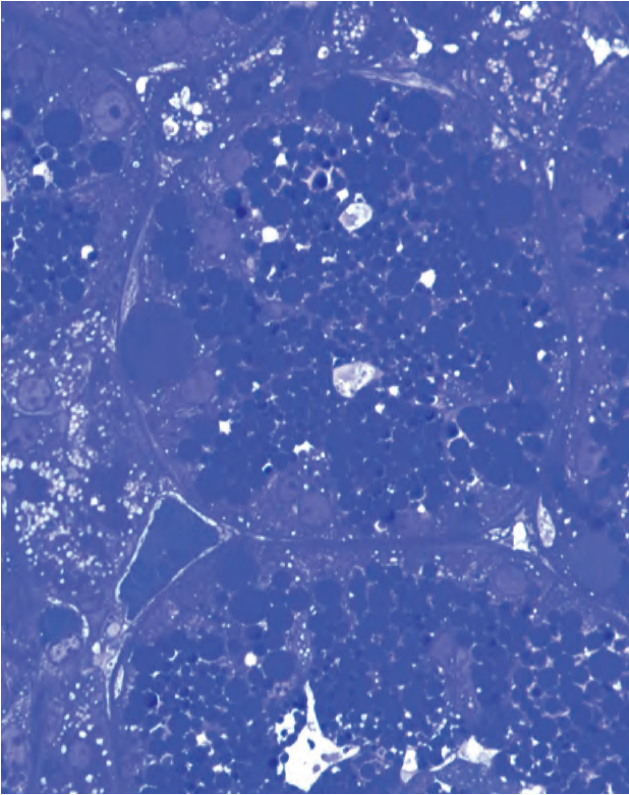

*Tpst2*<sup>-/-</sup>

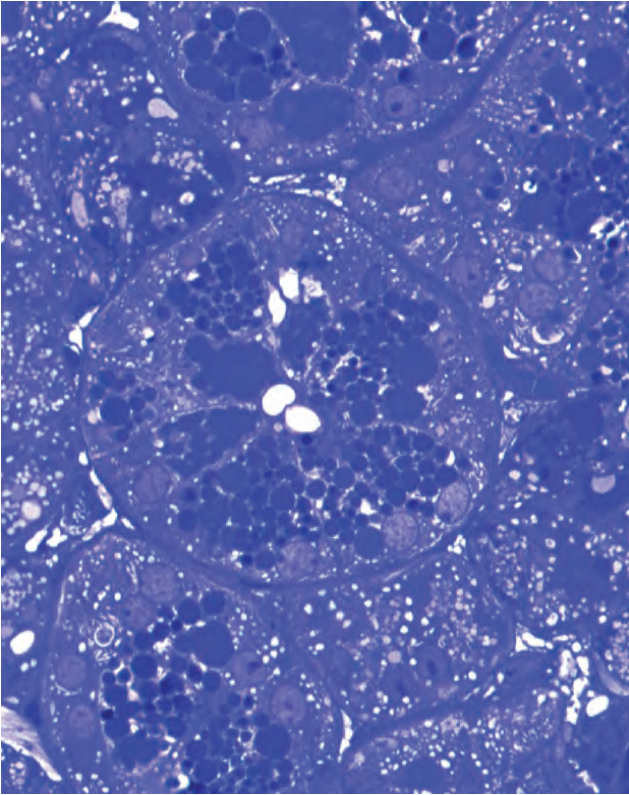

Thyroid diet

Supplement: Figure S5 — Submandibular gland histology on thyroid supplemented diet. At 15 weeks of age submandibular glands from female (top panels) or males (bottom panels) were harvested and then resin embedded, sectioned (0.5 µm) and stained with toluidine blue as described in Methods. Images are representative of analyses of the 4 mice randomly selected from the >10 mice in each experimental group in Figure 7. 63 x Objective. (PDF) [file pone.0071822.s005.pdf]
